# Reducing the bias of estimates of genotype by environment interactions in random regression sire models

**DOI:** 10.1186/1297-9686-41-30

**Published:** 2009-03-19

**Authors:** Marie Lillehammer, Jørgen Ødegård, Theo HE Meuwissen

**Affiliations:** 1 Department of Animal and Aquacultural Sciences, Norwegian University of Life Sciences, N-1432 Ås, Norway; 2 NOFIMA, N-1432 Ås, Norway

## Abstract

The combination of a sire model and a random regression term describing genotype by environment interactions may lead to biased estimates of genetic variance components because of heterogeneous residual variance. In order to test different models, simulated data with genotype by environment interactions, and dairy cattle data assumed to contain such interactions, were analyzed. Two animal models were compared to four sire models. Models differed in their ability to handle heterogeneous variance from different sources. Including an individual effect with a (co)variance matrix restricted to three times the sire (co)variance matrix permitted the modeling of the additive genetic variance not covered by the sire effect. This made the ability of sire models to handle heterogeneous genetic variance approximately equivalent to that of animal models. When residual variance was heterogeneous, a different approach to account for the heterogeneity of variance was needed, for example when using dairy cattle data in order to prevent overestimation of genetic heterogeneity of variance. Including environmental classes can be used to account for heterogeneous residual variance.

## Introduction

Random regression models are widely used to describe effects that change gradually over a continuous scale, for instance in genotype by environment interaction studies, where the genotype effect is modeled as a function of the environment [1]. A common measurement of the interaction is the variance in the slope of the sire reaction norms, *i.e. *sire breeding values regressed on an environmental variable. The interaction is regarded as significant if the slope variance is significant [*e.g. *[2,3,1]].

For the estimation of genotype by environment interactions, both sire models or animal models are used, however sire models are computationally less demanding. Thus the sire model is preferred when the model is complex, the amount of data is large, or the analysis has to be repeated many times, as in QTL analyses in which testing many positions is necessary.

Performing genetic analyses with a sire model gives an estimate of the "sire-variance", which is one fourth of the genetic variance. The remaining genetic variance (3/4) is modeled through the residual term together with the environmental variance. When the genetic variance is heterogeneous because of genotype by environment interactions, the residual variance will also be heterogeneous since part of it is genetic. Therefore, a random regression model that also accounts for heterogeneous residual variance is preferred [4,1].

One way to account for heterogeneous residual variance over environments is to divide the environment into classes and to assume homogeneous variance within each environmental class, but with different residual variances across classes [1]. The drawbacks of this method are that classes have to be arbitrarily defined and that the number of classes increases with the number of parameters that need to be estimated [5]. A more advantageous approach would be to model the residual variance as a function of the environment in the mixed model, but commonly used software does not facilitate this option [6]. Another possibility would be to add an extra term in the model, with a variance equal to three times the sire variance, which would model the part of the residual variance that is heterogeneous because of genetic heterogeneity. This term would be especially designed to capture residual variance originating from the genetic variance not modeled by the sire-term, but would not cover the heterogeneity of residual variance due to other origins.

The aim of this study was to compare available random regression models with regards to their ability to give unbiased estimates of genotype by environment interactions. Two animal models were compared to four sire models that differed in the modeling of residual variance. To test the models' ability to account for the heterogeneity of variance, two kinds of data were analyzed. Simulated data were generated to contain heterogeneous genetic variance, but homogeneous residual variance. In addition, dairy cattle data, in which both genetic and residual variances were assumed heterogeneous, were used to test the ability of the different models to model the variance heterogeneity.

## Methods

### Statistical models

Animal models and sire models differ in that animal models only model non-genetic variance in the residual term, while sire models also model part of the genetic variance in the residual term. Three classes of models were compared in this study. In addition to regular sire models and animal models, we applied sire models extended with a term to capture the remaining genetic variance not modeled by the sire-term. Within each of these classes of models, a model assuming homogeneous residual variance was compared to a model accounting for heterogeneous residual variance through the inclusion of environmental classes. All models are described below.

#### Animal models

The animal models are described by *y*_*i *_= *FIX *+ *a*_0*i *_+ a_1*i*_*env*_*i *_+ *e*_*i*_, where *y*_*i *_is the phenotypic value of daughter *i*, *FIX *is the fixed effects, which includes only the overall mean in the simulated data and a fixed regression on *env *in addition to the overall mean in the real data, *a*_0*i *_is the genetic effect of animal *i *on the intercept, *a*_01 _is the genetic effect of animal *i *on the slope, $Var(a)=A⊗\left[\begin{array}{cc} \sigma_{a0}^{2} & \sigma_{a0,a1}\\ \sigma_{a0,a1} & \sigma_{a1}^{2} \end{array}\right]$, where **A **is the relationship matrix among the animals, σ^2^_a0 _and σ^2^_a1 _are the genetic variances of the intercept and slope, respectively and σ_a0, a1 _is the genetic covariance between the intercept and slope. *env*_*i *_is the environmental value (herd-year effect in the real data) of daughter *i*, and *e*_*i *_is the residual, assumed either normally distributed with variance σ^2^_e _(animal-HOM), or homogeneous within each of 5 (simulated data) or 20 (dairy cattle data) environmental classes but varying between the classes (animal-CLASS): Var(e) = **X'DX**, where **X **is the design matrix that assigns the observations to different environmental classes, and $D=Diag\{\sigma_{e_{i}}^{2}\}$, where *i *≤ the number of environmental classes. Which environmental class an observation belongs to is dependent on its simulated environmental value (simulated data) or estimated herd-year effect (real data). The definition of the environmental classes is described in more detail in the paragraph on statistical analysis.

#### IND and IC sire models

Sire models, IND and IC, include an individual daughter term to account for the heterogeneous genetic variance not modeled in the sire term. The IC sire model also includes environmental classes that account for the heterogeneous residual variance and is expected to perform similarly to the animal-CLASS model. The IND sire model is expected to perform similarly to the animal-HOM model. The models are described by:

*y*_*i *_= *FIX *+ *S*_0*i *_+ *S*_1*i*_*env*_*i *_+ *ind*_0*i *_+ *ind*_1*i*_*env*_*i *_+ *e*_*i*_

where *y*_*i*_, *FIX *end *env*_*i *_are described as in the animal models,

*s*_0*i *_and *s*_1*i *_are the 1^st ^and 2^nd ^random regression coefficients of the sire of daughter *i*, $Var(s)=A_{s}⊗\left[\begin{array}{cc} \sigma_{s0}^{2} & \sigma_{s0,s1}\\ \sigma_{s0,s1} & \sigma_{s1}^{2} \end{array}\right]$, where **A**_**s **_is the relationship matrix among the sires, σ^2^_s0 _and σ^2^_s1 _are the sire variances of the intercept and slope, respectively and σ_s0, s1 _is the sire covariance between the intercept and slope. *ind*_0*i *_and *ind*_1*i *_model the effect of each individual from the intercept and slope respectively, as a deviation from the sire effect modeling the dam and Mendelian sampling effect. The variances of *ind *and *s *are constrained such that: $Var\left(\begin{array}{c} ind_{0}\\ ind_{1} \end{array}\right)=3\times Var\left(\begin{array}{c} s_{0}\\ s_{1} \end{array}\right)$. This restriction prevents over-parameterization of the model and inclusion of *ind*-terms in the model to increase the number of variance estimates. *e*_*i *_is the residual, either assumed normally distributed with variance σ^2^_e _as in the animal-HOM model (IND), or with Var(e) = **X'DX **as in the animal-CLASS model (IC).

#### HOM and CLASS sire models

The HOM and CLASS sire models omit the individual daughter term and are described by:

*y*_*i *_= *FIX *+ *S*_0*i *_+ *S*_1*i*_*env*_*i *_+*e*_*i*_, where all terms are defined as above. The HOM sire model assumes a homogeneous residual variance (as animal-HOM and IND), while the CLASS model uses environmental classes to account for the heterogeneous residual variance (as animal-CLASS and IC).

### Data

#### Simulations

Data were simulated with a heterogeneous genetic variance over an environmental scale and a homogeneous residual variance. The genetic effect of each animal was simulated and varied linearly with environment, which implies that the genetic effect was modeled by an intercept and a slope (the latter models the change of the genetic effect as environment changes). A base generation and three subsequent generations of animals were simulated. Generation 0 consisted of 100 unrelated animals, 50 males and 50 females, with random sampled genetic values for intercept (~N(0,0.3)) and slope (~N(0,0.016)). The genetic covariance between the intercept and slope was 0.06. Subsequent generations had breeding values drawn from the same distribution. Generation 1 consisted of 110 animals, 10 males and 100 females, produced from random mating of parents from generation 0. Generation 2 consisted of 500 males created by random mating of the parents in generation 1, and 50 000 unrelated females with randomly sampled genetic values. Generation 3 consisted of 50 000 daughters of the animals in generation 2, giving each male 100 offspring and each female 1 offspring. All animals in generation 3 were attributed, in addition to genetic values, an environmental gradient env~N(0,1), and a phenotypic value calculated as:

*y*_*i *_= a_0*i *_+ a_1*i*_*env*_*i *_+ *e*_*i*_, where a_0*i *_is the genetic value of intercept of animal *i *(σ^2^_a0 _= 0.3), *a*_1*i *_is the genetic value for slope of animal *i *(σ^2^_a1 _= 0.016, σ_a0a1 _= 0.06), *env*_*i *_is the environmental gradient of animal *i *(env ~ N(0,1)), and *e*_*i *_is a random residual e~N(0,0.5). The heritability of the average environment was 0.375. As a result of the model used for simulations, heritability increased with increasing environmental gradient.

The pedigree, phenotypes and environmental gradients of all animals in generation 3 were assumed known for the subsequent statistical analyses. The simulation was repeated 100 times.

#### Real data

Data of the first lactation protein yield from 604 637 daughters of 734 sires were obtained from GENO breeding and AI association (the Norwegian breeding association for dairy cattle). The data were pre-corrected for heterogeneous variance due to parity and age within parity, for the fixed effects of age within parity, month of calving within parity, days open within parity, year of calving and for the random effect of herd-year. These effects were estimated with the models used in the official Norwegian breeding value estimation. The estimated random effects for herd-year were used as the environmental descriptor (*env*) in the statistical analyses. All dams of daughters where assumed unrelated when creating the relationship matrix (**A**), used in the animal models, since female relationships were unknown.

### Statistical analysis

All statistical analyses were performed with the ASREML package [7]. The dairy cattle data were analyzed using all six models, while the animal-CLASS and sire IC models were omitted when analyzing simulated data. Since the simulated data did not include heterogeneous residual variance, these models were not believed to perform better than the corresponding models with homogeneous residual variance.

The environmental classes for the simulated data were defined with environments <-1.5 in class 1, environments ≥-1.5 and <-0.5 in class 2, environments ≥-0.5 and <0.5 in class 3, environments ≥0.5 and <1.5 in class 4 and environments ≥1.5 in class 5. For the dairy cattle data, the environmental classes were defined with 5 kg of protein within each class in environments between -45 and 45, and with one class capturing all environments below -45 and one class capturing all environments above 45. The environmental range between -45 and 45 captured 97.6% of the observations.

## Results

### Simulated data

Total genetic variance is modeled by three components: genetic variance of intercept, genetic variance of slope and genetic covariance between intercept and slope. Both the animal-HOM model and the sire model that includes an *ind*-term to account for 3/4 of the genetic variance (IND) gave unbiased estimates of all components (Table 1). This result was expected, since the dams were assumed unrelated, making the animal model and the IND-model equivalent. The sire model with homogeneous residual variance (HOM) and the sire model with classes of environments (CLASS) overestimated all genetic variance components. The use of classes of environments to account for the heterogeneous residual variance (CLASS) slightly reduced the bias of the genetic correlation between slope and intercept, but had little impact on the other genetic variance components.

**Table 1:** Genetics variance components and restricted maximum log-likelihood values in the simulated data, estimated by the different models

Model	Corr intercept-slope^a^	Intercept variance^a^	Slope variance^a^	Average REML^b^
Simulated value	0.866	0.300	0.016	
Sire model (HOM)	0.937_0.044_	0.324_0.025_	0.023_0.004_	0
Sire model (CLASS)	0.910_0.050_	0.325_0.025_	0.022_0.004_	167
Sire model (IND)	0.858_0.048_	0.298_0.017_	0.018_0.002_	178
Animal-HOM	0.858_0.048_	0.298_0.017_	0.018_0.002_	178

^a ^Standard deviations are given as subscripts.

^b ^Restricted maximum log-likelihood relative to the HOM sire model.

Average over 100 replicates

**Figure 1:**
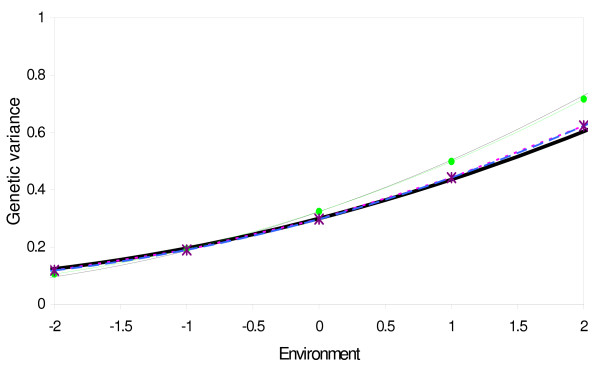
**Total genetic variance as a function of environment, estimated with the models HOM (thin black line), CLASS (green), IND (purple) and animal (blue), compared to the true simulated variance (thick black line)**.

Both animal-HOM and IND models gave approximately unbiased estimates of the total genetic variance over the environmental scale (Figure 1), while sire HOM and CLASS models gave a slight underestimation of total genetic variance in the lowest environments and an overestimation in the highest environments. The average log-likelihoods from the different models over 100 replicated simulations are reported in Table 1. Animal-HOM and IND models gave the highest log-likelihood values, showing that they are the best suited to model heterogeneous genetic variance.

All the sire models were computationally much faster than the animal models. The sire models needed respectively 2% (HOM), 5% (CLASS) and 4% (IND) of the computational time required for the animal-HOM model.

### Real data

The log-likelihoods of the different models are reported in Table 2. The highest log-likelihood was obtained with model IC, which combines the use of an individual term and environmental classes, and has the same number of parameter estimates as the CLASS sire and animal-CLASS models.

**Table 2:** Log-likelihood-values from analyzing the dairy cattle data

Model	REML^a^
Animal-HOM	4027.4
Animal-CLASS	4145.6
Sire model (HOM)	0
Sire model (CLASS)	4132.1
Sire model (IND)	4032.3
Sire model (IC)	4147.6

^a ^Restricted maximum log-likelihood relative to the HOM sire model

**Figure 2:**
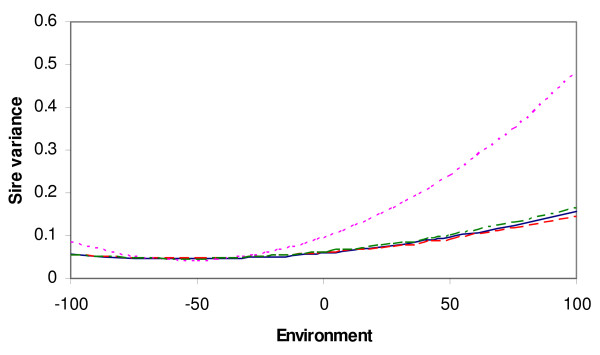
**Sire variance in the dairy cattle data, modeled as a function of an environmental parameter, estimated by the different models**. HOM (purple), CLASS (red), IND/animal-HOM (pink) and IC/animal-CLASS (green); two models are presented with the same line if their results are too similar to be distinguishable.

**Figure 3:**
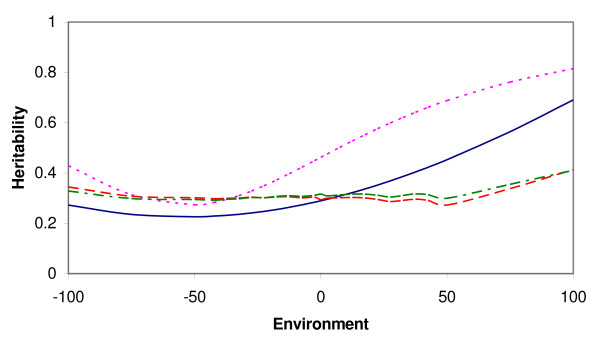
**Heritability in the dairy cattle data, over a range of environments, estimated by the different models**. HOM (purple), CLASS (red), IND/animal-HOM (pink) and IC/animal-CLASS (green); two models are presented with the same line if their results are too similar to be distinguishable.

Residual variance was found to be heterogeneous with all models able to capture heterogeneity of residual variance. All the models that included heterogeneous residual variance gave similar estimates of residual variance for the environmental range capturing most of the data. The sire variance was heterogeneous with all models, but much more variable with the IND and animal-HOM models than with the other models (Figure 2), which is probably due to the inability of animal-HOM and IND models to model residual heterogeneity of non-genetic sources. The heritability (Figure 3) seemed to be approximately constant over environments when modeled by a model that included environmental classes, while more variable when modeled by a model that did not include environmental classes. Animal-HOM and IND sire models gave very similar estimates of variance components. Similarly, the animal-CLASS model gave estimates very similar to the IC-model.

The HOM sire model seemed to underestimate the heritability in low-yield environments (due to an overestimation of residual variance in those environments), and to overestimate heritability in high-yield environments (where residual variance is underestimated). IND and animal-HOM models seemed to overestimate the heritability in high environments and to underestimate heritability over most of the low-yield environmental range, caused by a biased estimation of the genetic variance, which was inflated because these models did not account for heterogeneous residual variance.

Correlations between the sire breeding values obtained by the different models are reported in Table 3. The high correlations between breeding values obtained by the different models indicate that the ranking of animals is less affected by the choice of the model than the estimates of variances and covariances across environments.

**Table 3:** Correlations between breeding values estimated by the different models

	HOM	IND	CLASS	indCLASS	Animal-HOM	Animal-CLASS
HOM	1					
IND	0.975	1				
CLASS	0.996	0.965	1			
indCLASS	0.998	0.976	0.999	1		
Animal-HOM	0.975	1.000	0.965	0.976	1	
Animal-CLASS	0.998	0.976	0.999	1.000	0.975	1

## Discussion

Estimation of genotype by environment interactions by random regression sire models with homogeneous residual variance can result in biased estimates of the variance components (Fig. 1). Since 3/4 of the genetic variance is modeled in the residual term in the sire model, heterogeneous genetic variance causes the residual variance to be heterogeneous as well. When the sire variance is the only variance allowed to change across the environmental scale, overestimation of sire slope variance and/or genetic correlation between slope and intercept enable the model to capture some of the heterogeneity in residual variance. Consequently as expected, the sire model that assumes homogeneous residual variance (HOM), overestimated both genetic slope variance and genetic correlation between slope and intercept in the simulated data. However, in the real data the estimated sire-variances obtained by the HOM sire model are similar to those obtained by the models accounting for heterogeneous variance by environmental classes (Figure 2).

In the dairy cattle data, the residual variance seems to be more heterogeneous than expected from the genetic component. The models that provided approximately unbiased estimates when analyzing simulated data (IND and animal-HOM) probably caused an overestimation of genetic slope variance and genetic correlation between slope and intercept in the real data. The term corresponding to the animal (*ind *in the sire model and *a *in the animal model) is probably well suited to model the heterogeneity of residual variance, causing an increased log-likelihood, compared to HOM. Using the IND sire model, constraints in the model cause the sire-variance to be overestimated if the *ind*-term captures a larger part of the residual than 3/4 of the true genetic variance. The animal-HOM model also assumes that only the genetic variance can be heterogeneous, and thereby overestimates the heterogeneity of the genetic variance when other sources of heterogeneous variance are present. Hence, heterogeneity of residual variance, regardless of origin, should be accounted for, even in models including an *ind*-term or in animal models. IC and animal-CLASS models can do it.

Table 2 shows that the largest gain in log-likelihood when analyzing real data is obtained by fitting environmental classes, defending the increased number of variance components in the model. Using environmental classes to account for heterogeneous residual variance has the advantage that no assumption has to be made about the shape of the residual variance curve. However, the drawback is that the residual variance is assumed to change only at certain arbitrarily defined environmental values, rather than to follow a continuous curve.

The IND sire model gives a higher log-likelihood than the animal model (Table 3), and the variance components estimated by the two models are very similar but not equal. The same holds for the sire model IC versus the animal-CLASS model. Sire models containing an *ind*-term would be equivalent to animal models in cases where the females are unrelated (as in the simulated data) or unknown (like in the real data). The latter is only strictly true if the sires are non-inbred, because with inbred sires, the within sire genetic variance is expected to be slightly smaller than three times the sire variance, and the animal model accounts for this reduction in variance because of inbreeding. When the IND sire model gives a higher log-likelihood than the animal-HOM model and the IC model gives a higher likelihood than the animal-CLASS model, this implies that the true genetic variance is constant or increasing over generations instead of decreasing because of the accumulation of inbreeding. Differences between the animal models and the corresponding sire models are so small that the variance estimates between the models cannot be distinguished in the figures (Figures 2 and 3), and the correlations between breeding values from these models are approximately 1 (Table 3). When ignoring relationships between sires, animal-HOM and IND sire models give the exact same log-likelihood as well as the exact same variance components (result not shown). Genetic variance is often maintained over multiple generations of selection, even though, in theory, inbreeding should reduce genetic variance [8]. Animal models might give more unbiased estimates of variance components than sire models with *ind*-terms if female relationships were known and could be properly accounted for.

All sire models are more computer efficient as compared to animal models, which is important if the amount of data is large or if the analysis has to be repeated many times, as in QTL by environment interaction analyses [9]. In such cases, at least if female relationships cannot be accounted for, sire models with *ind*-terms should be preferred over animal models.

If we remove the constraint that the *ind*-variance is three times the sire-variance from the IND sire model, it could prevent overestimation of the sire-variance because of bias in the *ind*-term. However, this model would then be over-parameterized because the *ind*-term is allowed to absorb the residual term. ASReml has reported singularities in average information matrix when such an unconstrained IND sire model is fitted.

One of the benefits of replacing environmental classes (CLASS) with an *ind*-term (IND) is the reduction of the number of parameters in the model. Combining IND and CLASS in the IC model gives equally many parameters as CLASS, and the advantages of including the *ind*-term in addition to environmental classes can therefore be discussed. However, including an *ind*-term increases the log-likelihood significantly without increasing the number of parameters to be estimated by the model (Table 2); the IC model is more than 8 million times more likely than the CLASS model. The IC model gives a smoother estimate of the residual variance curve over environments, causing more accurate estimates of the residual variances close to the limits between the environmental classes. This is probably why this model fits the data better. Using the IC sire model gives a slightly higher heritability in high-yield environments and lower heritability in low-yield environments, compared to the CLASS sire model.

In cases where the residual variance is known to be homogeneous, including an *ind*-term could be useful to capture the part of the genetic variance not covered by the sire-term in the sire model. This might be useful for instance in survival models and analyses of categorical data, where residuals are often not explicitly included in the model and thus assumed to have homogeneous residual variance at the underlying scale.

## Conclusion

Using an individual term to model the genetic effect not covered by the sire-effect seems to be an adequate way to model heterogeneous residual variance caused by heterogeneity of genetic variance. However, in cases where heterogeneity in residual variance has other origins, these models may overestimate genetic variance. These problems are common to both sire models including an *ind*-term and the widely used animal models. Environmental classes can be used in these cases to capture the non-genetic part of the residual variance.

## Competing interests

The authors declare that they have no competing interests.

## Authors' contributions

ML participated in designing the study, developed the simulation program, performed simulations and statistical analyses and drafted the manuscript. JØ helped develop the statistical methodology and write the manuscript. TM participated in designing the study, supervised the study and participated in writing the manuscript.
